# The Potential of Extracellular Vesicle-Mediated Spread of Self-Amplifying RNA and a Way to Mitigate It

**DOI:** 10.3390/ijms26115118

**Published:** 2025-05-26

**Authors:** Maurizio Federico

**Affiliations:** National Center for Global Health, Istituto Superiore di Sanità, 00161 Rome, Italy; maurizio.federico@iss.it

**Keywords:** self-amplifying RNA, alphaviruses, SARS-CoV-2 vaccines, extracellular vesicles, HIV-1 Nef

## Abstract

Self-amplifying RNA-based (saRNA) technology represents the last frontier in using synthetic RNA in vaccinology. Typically, saRNA consists of positive-strand RNA molecules of viral origin (almost exclusively from alphaviruses) where the sequences of structural proteins are replaced with the open reading frame coding the antigen of interest. For in vivo delivery, they are complexed with lipid nanoparticles (LNPs), just like current COVID-19 vaccines based on synthetic messenger RNA (mRNA). Given their ability to amplify themselves inside the cell, optimal intracellular levels of the immunogenic antigen can be achieved by delivering lower amounts of saRNA molecules compared to mRNA-based vaccines. However, the excessive intracellular accumulation of saRNA may represent a relevant drawback since, as already described in alphavirus-infected cells, the recipient cell may react by incorporating excessive RNA molecules into extracellular vesicles (EVs). These EVs can shed and enter neighboring as well as distant cells, where the EV-associated saRNA can start a new replication cycle. This mechanism could lead to an unwanted and unnecessary spread of saRNA throughout the body, posing relevant safety issues. This perspective article discusses the molecular mechanisms through which saRNAs can be transmitted among different cells/tissues. In addition, a simple way to control the possible excessive saRNA intercellular propagation through the co-expression of an EV-anchored protein inhibiting the saRNA replication is proposed. Based on current knowledge, a safety improvement of saRNA-based vaccines appears to be mandatory for their usage in healthy humans.

## 1. Introduction

On 12 December 2024, the European Medical Agency (EMA)’s “Committee for Medical Products for Human Use” (CHMP) recommended the medicinal product Kostaive for approval [1]. On 12 February 2025, the European Commission, implementing the EMA’s indication, granted the authorization to its marketing [2]. Kostaive is the commercial denomination of the ARCT-154 vaccine [3,4], which, as in the case of mRNA-based vaccines, should be more appropriately defined as a pro-drug. It is a pharmaceutical product based on lipid vesicles containing self-amplifying RNA molecules encoding the stabilized Spike protein of SARS-CoV-2 and designed to protect against COVID-19 disease. Due to the ability to replicate in the target cell, lower doses of RNA are needed to achieve levels of immune responses similar to those induced by the injection of the widely diffused messenger RNA-based COVID-19 vaccines.

Besides ARCT-154, at least four additional COVID-19 products based on saRNA are under scrutiny in clinical trials, including COVAC1 [5,6,7] and GEMCOVAC-OM [8], both expressing full-length, stabilized SARS-CoV-2 Spike, and VLPCOV-1 [9], along with its improved version, VLPCOV-2 [10], which express the Spike receptor-binding domain. As for ARCT-154, these products are derived from the genome of the Venezuelan Equine Encephalitis virus and are encapsulated into synthetic lipid nanoparticles similar to the currently available mRNA-based COVID-19 vaccines. Differently from the latter, however, none of the saRNA-based products incorporate the 5′-methyl pseudouridine in their RNA sequences, given its inhibitory effects on the saRNA replication [11]. The saRNA-related technology was also the basis for the production of vaccines against the Rabies virus, which are currently being tested in clinics [12].

From a technological point of view, the development of drugs and vaccines based on saRNA undoubtedly represents a breakthrough. Its use in humans followed a few years after the rollout of mRNA-based COVID-19 vaccines, which in turn represented an important innovation. As in the case of mRNA-based technology, saRNA-based drugs and vaccines are expected to be experimented with and applied in different fields, from infectious to tumor diseases. However, relevant safety issues still need to be addressed, especially regarding the use of saRNA expressing biologically active products in healthy humans, also considering that current rules for nonclinical evaluation of vaccines do not require pharmacokinetic studies [13]. In this perspective article, the molecular mechanisms at the basis of the saRNA activity and its interaction with the intracellular sorting machinery are summarized. Unexplored safety issues are also depicted, together with a theoretical way to control them. Optimizing the safety of new biotechnologies proposed for healthy humans is a mandatory issue.

## 2. The saRNA Replication Cycle

The saRNA-based technology relies on the engineering of the genome of alphaviruses, i.e., positive-strand RNA viruses, in particular Venezuelan Equine Encephalitis virus, Semliki Forest virus (SFV), and Sindbis virus [14]. Upon cell entry, saRNA molecules can amplify themselves while expressing quite high levels of the gene of interest, which is instrumental, in many instances, to induce a strong antigen-specific immunity.

**Figure 1:**
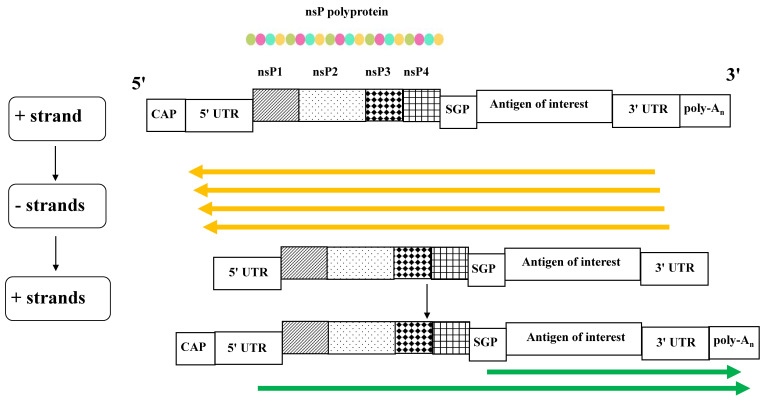
Scheme of the saRNA replication. Upon cell entry, the sequences for the non-structural proteins nsP1–P4 are translated, generating a polyprotein complex which, upon partial self-cleavage, synthesizes the complementary, negative RNA strands (in yellow). They serve as templates to generate both genomic and sub-genomic messenger (m)RNAs (in green), the latter specifically devoted to the production of the antigen of interest. CAP: 5′ cap structure; UTR: untranslated region; SGP: sub-genomic promoter; poly-A: polyadenylated tail.

In the alphavirus genome, nonstructural, replicative proteins are coded by sequences located at the 5′ end, and sequences at the 3′ end code the structural proteins. The amplification of saRNA, which overlaps the alphavirus replication cycle [15], begins with the translation of the non-structural nsP1-P4 proteins. They form a polyprotein complex which, upon partial cleavage, synthesizes the complementary, negative RNA strands that serve as templates to generate both genomic and sub-genomic messenger (m)RNAs. The latter are specifically devoted to the production of the antigen of interest (Figure 1).

The functions of each of the four non-structural proteins have been investigated in depth [16]. NsP1 is a capping enzyme that anchors the viral replicase complex to the cell membranes. NsP2 has a helicase function, a protease activity, and is involved in the virus RNA packaging. NsP3 interacts with several host cell proteins, and its inactivation drastically reduces the genome replication efficiency and the sub-genomic RNA expression, thus affecting viral fitness. Finally, nsP4 has RNA-dependent RNA polymerase (RDRP) activity.

To produce the desired immunogen, the alphavirus genome is engineered so that the open reading frames coding for structural proteins are replaced with sequences specific for the gene of interest, i.e., those of SARS-CoV-2 Spike in the case of saRNA-based COVID-19 vaccines. In this manner, the gene of interest is translated in the late phase of the replication cycle by sub-genomic RNAs whose expression is regulated by an internal, sub-genomic promoter.

The most evident advantage of saRNA over the mRNA-based technology is represented by the lower amounts of RNA molecules to be administered to achieve a comparable immune response. For instance, levels of the immune response similar to those generated by the inoculation in mice of an mRNA vaccine were obtained by a more than 60-fold lower dose of saRNA [17]. In the phase 3 clinical trial, the inoculation of 5 µg-RNA equivalents of saRNA produced immunogenic effects of similar strength to those elicited by 30 µg of an mRNA-based vaccine [4]. From a biological point of view, the most striking difference is that, whereas the artificial mRNA, once entered into the cell, can either persist, supported by the TENT5A-induced re-adenylation [18], or gradually degrade, saRNA can reproduce itself and accumulate inside the target cell.

## 3. The Intracellular Fate of saRNA and Its Loading into Extracellular Vesicles

The most relevant biological feature of saRNA molecules consists of their ability to replicate themselves once internalized by target cells. The ultimate products of the replication cycle are positive-strand, full-length RNA molecules stabilized by a 5′ cap and polyadenylated at their 3′ end, together with sub-genomic mRNAs which, upon polyadenylation, become templates for the production of the antigen of interest.

Different from the replication cycle of the parental virus, where neo-synthesized, full-length RNA assembles with the neo-synthesized, structural viral proteins to form the viral progeny, neo-synthesized full-length saRNA is expected to accumulate intracellularly while resisting rapid intracellular degradation. Data from the literature help in anticipating the fate of neo-synthesized saRNA molecules. In particular, relevant results were obtained considering the sophisticated mechanisms that cells activate to remove the excess of extraneous molecules, in particular the multivesicular body/exosome system [19,20].

All cells constitutively release vesicles of various sizes, recognizing different biogenesis [21]. Extracellular vesicles (EVs) released by healthy cells are generally distinguished into microvesicles (50–1000 nm) and exosomes (50–200 nm). Both microvesicles (also referred to as ectosomes) and exosomes are lipid bilayer vesicles. The former are shed from the plasma membrane, whereas the latter originate intracellularly from the inward invagination of endosome membranes. This process induces the formation of intraluminal vesicles (ILVs), which become part of multivesicular bodies (MVBs). They can traffic either to lysosomes for degradation or to the plasma membrane, to which they fuse, thereby releasing their contents in the extracellular milieu as exosomes.

Originally, EVs were thought to be garbage bags through which cells eject their waste. Today, it is widely accepted that EVs are also key components of the intercellular communication network. They incorporate mRNAs, microRNAs (miRNAs), DNA, and proteins, which can be functional in target cells [22]. Due to their stability in biological fluids, EVs can circulate in the body, and their interaction with target cells can lead to their internalization. It is mediated by a wealth of mechanisms, including binding to specific cell receptors and fusing with the plasma membrane, followed by the delivery of exosome cargo directly to the cytoplasm, micropinocytosis, phagocytosis, and endocytosis mediated by either clathrin, caveolin, or lipid rafts.

Concerning their molecular composition, some EV proteins are cell-type-specific, while others are invariable parts of EVs independently of the cell of origin. Typical proteins found in microvesicles are CD40, selectins, integrins, and cytoskeletal proteins. On the other hand, exosomes are enriched with products involved in MVB formation (e.g., Alix, TSG101), membrane transport and fusion (e.g., annexins, flotillins, GTPases), adhesion (e.g., integrins), tetraspanins (e.g., CD9, CD63, CD81, CD82), and antigen presentation (MHC class I and II molecules).

EVs can carry both short and long RNAs. Besides mRNAs and miRNAs, other RNA species have been found in EVs, such as viral RNAs, Y-RNAs, fragments of tRNAs, mitochondrial RNA, small nuclear RNA, small nucleolar RNA, piwi-interacting RNAs, and long non-coding RNAs [23]. The mechanisms governing the specific loading of RNA species into EVs are only partly known. The EV loading of RNA occurs through either active or passive mechanisms. In the former context, RNA-binding proteins (RBPs) play a key role in sorting RNA molecules into exosomes [24,25]. A short nucleotide motif regulating the sorting of RNA into exosomes through binding with the ubiquitous heterogeneous nuclear RNP-A2B1 has been identified [26]. Afterward, an alternative short nucleotide sequence has been detected as the binding motif for the hnRNP Q-mediated delivery of miRNAs into exosomes released by hepatocytes [27]. Together, these sequences are part of the so-called “exomotifs”, which play an essential role in active RNA loading in exosomes [28].

On the other hand, RNAs can be loaded into EVs by passive mechanisms driven by the high intracellular concentration of a specific RNA [29]. This could be the case of neo-synthesized, full-length saRNA molecules whose intracellular accumulation is expected to be as high as that following acute virus infection.

## 4. EVs as Vehicles of Propagation of the Alphavirus Genome: The Potential of EV-Associated saRNA Spread

Both in vitro and in vivo studies demonstrated the spread of the genome of alphaviruses through EVs. In detail, it was reported that both Semliki Forest virus and Sindbis virus genomes defective for the expression of capsid proteins can propagate in both mammalian and insect cells through EVs [19]. These defective genomes propagate in the presence and the absence of the co-expression of the respective Spike proteins. The EVs emerging from the cells expressing the mutated viral genomes were shown to incorporate the replication-competent, positive-strand viral RNA and were infectious in vivo, where they spread most efficiently in the lungs. Similar conclusions were drawn by analyzing the supernatants of epithelial cells infected with another alphavirus, i.e., the Chikungunya virus [20].

**Figure 2:**
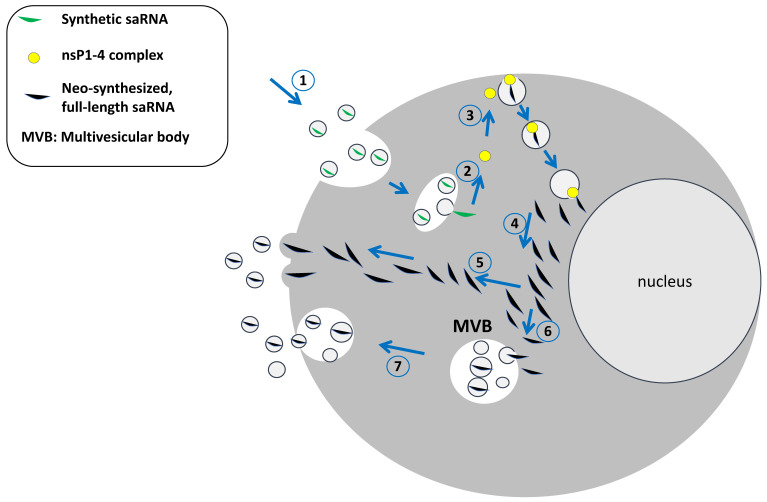
A model of the intracellular fate of saRNA. After the intracellular delivery driven by LNPs to which saRNAs are complexed (1), the replication cycle switches. After the release of saRNA into the cytoplasm (2), the replication cycle driven by the neo-synthesized nsP1-4 protein complex takes place in protected sites called “spherules,” where saRNA accumulates (3). Both genomic and sub-genomic positive saRNA strands are then delivered to the cytoplasm (4). In the absence of structural virus proteins with which to interact, cap-stabilized genomic saRNA molecules can be sorted into microvesicles emerging from the plasma membrane (5), as well as into intraluminal vesicles accumulating in MVBs (6), which are finally released into the extracellular space (7). The ultimate result is the shedding of saRNA-incorporating EVs.

Based on these consistent experimental pieces of evidence, it appears more than conceivable that similar events occur in cells entered by saRNAs (Figure 2). EVs emerging from these cells can enter neighboring as well as distant cells and tissues, and the spread of saRNA-loaded EVs can lead to a viral-like expansion. The EV-mediated spread of saRNA might also be favored by the direct uploading in exosomes of LNP-saRNA molecules escaping the endosomal degradation as described for both erythropoietin- and VEGF-A-expressing mRNAs [30,31].

While these mechanisms may be somewhat advantageous regarding the desired immunogenicity, they may be considered virtually off-target processes. In fact, unlike most virus species, EVs can enter the cells of any tissue/organ, given their multiple mechanisms of cell entry.

In this scenario, the only hindrance against the spread of saRNA-EVs would be the adaptive immune response elicited against the antigens expressed by the saRNA.

However, both humoral and cellular immune responses need days to mount efficiently, while the saRNA replication cycle is expected to be completed in hours, and EVs can diffuse in minutes.

Additional results from biodistribution studies support the idea that saRNA can have replicative potential in vivo. A single intramuscular injection of saRNA expressing the Rabies glycoprotein in rats led to the distribution of the vaccine in the lungs, liver, and spleen within two days. Significantly, the saRNA load detected in the lungs increased more than one hundred-fold at day fifteen post-injection. Strong increases of saRNA levels have also been documented in both the liver and spleen eight days after the inoculation [32].

In another biodistribution study, the amounts of avian influenza virus-hemagglutinin expressing saRNA detected in the spleen of the injected mice increased from day 5 to day 7 after the intramuscular administration [33]. Taken together, these results strongly corroborate the earlier evidence obtained with defective SFV and Sindbis virus genomes.

The expected consequences of the saRNA spread mostly rely on the biological activity of the expressed gene of interest. The case of full-length, stabilized SARS-CoV-2 protein needs some specific considerations. First, the protracted presence of Spike, mainly a consequence of vaccine mRNA persistence, has been documented in vaccinees [34,35], thus suggesting that the immune response cannot rapidly eliminate the cells expressing the SARS-CoV-2 Spike protein. Second, it has been suggested that SARS-CoV-2 Spike protein associates with exosomes [36,37]. In such an instance, it should be investigated whether Spike-expressing exosomes can be facilitated to enter and deliver saRNA molecules in ACE2-expressing cells, and the consequences thereof. Finally, and likely most importantly, the effect of the expression of SARS-CoV-2 Spike protein diffused through the body should be evaluated in terms of its overall toxicity consequence of the binding with ACE2, as well as additional molecular targets [38], leading to unwanted effects including inflammatory responses, immune dysregulation, and autoimmunity [39,40,41,42].

In any case, a peculiar feature of the saRNAs is their potential ability to spread into the body. Hence, looking for a method to mitigate/inhibit their uncontrolled spread appears largely desirable.

## 5. A Way to Control the saRNA Spread

The uncontrolled circulation of EVs incorporating full-length RNA can represent a safety limitation for the usage of saRNA-based vaccines in humans. To overcome such a potential drawback, the co-expression in EVs of an inhibitor of the saRNA replication would be of great help. On this subject, we identified an HIV-1 Nef-defective protein mutant, i.e., Nef^mut^, acting as an EV-anchoring protein [43]. It is a functionally defective protein mutant lacking the Nef effects typically associated with HIV pathogenesis and showing an extraordinary ability to incorporate into EVs, i.e., from 50 to 100-fold more efficiently than the wild-type isoform. Nef^mut^ can be fused at its C-terminus to proteins of choice, meanwhile retaining its EV-anchoring properties. Therefore, Nef^mut^ allows the incorporation of high amounts of antigens fused to it into EVs, which, thus, remain protected from external neutralization and/or degradation factors. Nef^mut^ binds to the inward leaflets of both intracellular and plasma membranes to which it tightly interacts through both its N-terminal myristoylated and palmitoylated tails [44].

**Figure 3:**
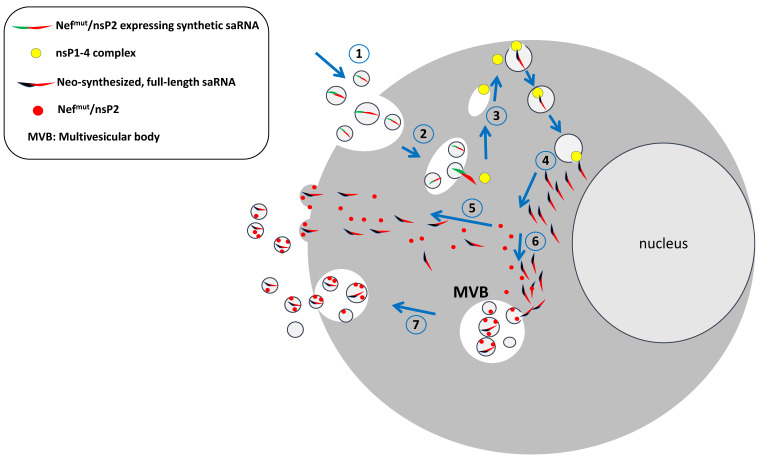
Generation of self-limiting saRNA. After the cell entry and the replication cycle completion (1–4), the translation of sub-genomic RNA molecules leads to the production of Nef^mut^/nsP2. The fusion product reaches both the internal side of the plasma membrane (5) and the intraluminal vesicles (6–7), thereby being incorporated into emerging EVs together with full-length saRNA.

Cells infected by alphaviruses resist the homologous superinfection through a block occurring at the level of viral RNA transcription [45,46]. It has been reported that the co-expression of nsP2 inhibits the activity of the homologous RDRP [47]. Based on this experimental evidence, the characteristics of Nef^mut^ would be instrumental to achieving the control of saRNA spread. In particular, a modified saRNA would be designed in a way that a Nef^mut^/nsP2 fusion protein is co-expressed with the antigen of interest through the creation of a bi-cistronic RNA by joining the respective sequences with an internal ribosome entry site (IRES) [48]. In this way, cells internalizing the saRNA accumulate the Nef^mut^/nsP2 fusion products into their nascent EVs. Therefore, when the saRNA-incorporating EVs enter bystander cells, the replication cycle of saRNA can be limited by the inhibitory effect of EV-associated nsP2 (Figure 3).

In addition, a large body of experimental evidence demonstrated that EVs incorporating antigens fused with Nef^mut^ induce a strong CD8^+^ T lymphocyte cytotoxic (CTL)-driven immune response leading to the elimination of the antigen-expressing cells [49,50,51]. Hence, EVs emerging from Nef^mut^/nsP2 expressing cells can also act as a specific immunogen able to elicit a CTL immune response against saRNA-expressing cells. In this way, the immune response against Nef^mut^/nsP2, which, given its overexpression, is expected to be prevalent compared to that towards the other alphavirus proteins, would contribute to controlling the saRNA spread.

In sum, co-expressing Nef^mut^/nsP2 in the context of saRNA-based vaccines is expected to protect from unwanted/unexpected side effects due to EV-mediated, uncontrolled saRNA spread. This protection is assumed to occur through two distinct mechanisms, i.e., by inhibiting the saRNA replication in bystander cells and inducing CTL immunity against nsP2-expressing cells. This strategy would specifically mitigate the risks related to self-amplification. In the case of Spike-based COVID-19 vaccines, the issues of how much Spike protein can be produced and how long for would need to be investigated.

## 6. Conclusions

The development of the saRNA-based technological platform certainly opens quite interesting perspectives in basic, translational, pre-clinical, and clinical research. As already occurred with the retro- and lentivirus-based technologies, deep knowledge of the virus biology allowed the manipulation of their genomes with the ultimate aim of creating new preventive/therapeutic drugs. For instance, lentiviral vectors are exploited to produce CAR-T cells to cure oncologic patients [52], while a saRNA-based COVID-19 vaccine has been commercialized for use in healthy persons [1]. This fact imposes an accurate evaluation of the potential biological risks.

The results from the phase 3 clinical trial of ARCT-154 given as a fourth-dose booster after three doses of an mRNA-based vaccine suggest that its protection efficacy is not inferior to that induced by the fourth dose of the mRNA vaccine considered as a benchmark [4]. However, the actual impossibility of evaluating the immunologic consequences of previous anti-SARS-CoV-2 immunizations renders the results difficult to interpret.

Apart from the not-so-obvious advantages of this new generation of COVID-19 vaccines, the use of saRNA in healthy humans poses unprecedented safety issues that have been only partially investigated. Hick and coll. demonstrated a reduced replication of homologous alphaviruses in cells expressing saRNAs given the effects of homologous viral interference [53]. This mechanism reduces the possibility of recombination between the infecting virus and the saRNA, although the block appears to be incomplete, and some co-replication is still possible depending on the virus/saRNA doses used and the timing of superinfection. On the other hand, no viral recombinations have been detected in mice injected with saRNA and infected with the parental alphavirus.

Conversely, nothing is known about the possible spread of saRNA molecules. Here, a realistic mechanism of saRNA intercellular transmission based on previous experimental findings is proposed. A peculiar feature of saRNA molecules is their efficiency in replicating themselves, just as virus genomes do. However, different from the replication cycle of authentic viruses, full-length saRNA molecules are expected to accumulate intracellularly since they cannot egress the cell upon association with the viral structural proteins. Notably, and unlike many other virus species, the genome of alphaviruses efficiently sheds into the emerging viral particles [54] and, as demonstrated for the Chikungunya virus [20], also in EVs.

For these reasons, investigating whether the intracellular accumulation of full-length saRNA associates with the generation of saRNA-incorporating EVs appears to be mandatory. The association of viral RNA with EVs is not a novelty in the virology field. For instance, lentiviruses exploit the exosome intercellular traffic for both the biogenesis of viral particles and as a way of infection [55]. Similarly, transmission through EVs has been described for HBV [56], HCV [57], HSV [58], and the Dengue virus [59].

A deep investigation on the possible association of saRNA with EVs is also urgent, considering the recently commercialized vaccine designed to express SARS-CoV-2 Spike, i.e., a biologically active protein able to bind and activate the widespread ACE2 cell receptor. The excessive redistribution of Spike-expressing saRNA may exacerbate the adverse events already described for mRNA-based vaccines [38], as well as increase the number of cells that can be attacked and killed by the evoked anti-Spike immune response. It was reported that the expression of the viral envelope protein (i.e., Spike) is not necessary for the replication of the alphavirus genome incorporated into EVs [19]. However, the association of SARS-CoV-2 Spike with these EVs is expected to facilitate their delivery in ACE2-expressing cells, thus rendering the overall scenario even more complicated.

Some additional facts call for an urgent investigation of the possible saRNA-EV association. First, many authors demonstrated that circulatory EVs can readily migrate in lung tissues [60]. On this subject, EVs associated with the capsid-defective SFV genome have been found to replicate in the lungs quite efficiently, still much better than the wild-type virus [19]. Second, well-detectable amounts of EVs have been found associated with lung exhalations [61,62,63]. Therefore, besides body fluids, lung exhalations might be a way of transmitting the saRNA-incorporating EVs, while opening the theoretical possibility of an environmental impact [64]. Third, EVs do not recognize effective species barriers.

The proposed strategy of inactivation of the saRNA transmission would be a way to mitigate the risk of unwanted overexpression of the gene of interest to be exploited for the design of second-generation saRNA-based vaccines in an effective bi-cistronic configuration.

## Data Availability

No new data were created.
